# Fecal and oral microbiome analysis of snakes from China reveals a novel natural emerging disease reservoir

**DOI:** 10.3389/fmicb.2023.1339188

**Published:** 2024-01-11

**Authors:** Xiaoliang Hu, Lu Yang, Yue Zhang, Manman Yang, Jiayi Li, Yuping Fan, Peng Guo, Zhige Tian

**Affiliations:** Yibin Key Laboratory of Zoological Diversity and Ecological Conservation, Faculty of Agriculture, Forestry, and Food Engineering, Yibin University, Yibin, China

**Keywords:** *Protobothrops mucrosquamatus*, *Elaphe dione*, *Gloydius angusticeps*, microbiome, bacterial pathogens

## Abstract

**Introduction:**

The gastrointestinal tract and oral cavity of animal species harbor complex microbial communities, the composition of which is indicative of the behavior, co-evolution, diet, and immune system of the host.

**Methods:**

This study investigated the microbial composition in snakes from varying altitudinal ranges by assessing the fecal and oral bacterial communities in *Protobothrops mucrosquamatus, Elaphe dione*, and *Gloydius angusticeps* from Sichuan Province, China, using metagenomic sequencing.

**Results and discussion:**

It was revealed that Bacteroidetes, Proteobacteria, Firmicutes, and Fusobacteria were the core microbial phyla in fecal samples across all three species, while Proteobacteria, Bacteroidetes, Actinobacteria, and Firmicutes were the core microbial phyla in oral samples across all three species. Notably, the dominance of Armatimonadetes was documented for the first time in the feces of all three species. Comparative analysis of the microbiomes of the three species indicated distinct microbiological profiles between snakes living at low- and high-altitude regions. Furthermore, 12 to 17 and 22 to 31 bacterial pathogens were detected in the oral and fecal samples, respectively, suggesting that snakes may serve as a novel reservoir for emerging diseases. Overall, this study provides a comparative analysis of the fecal and oral microbiomes in three snake species. Future investigations are anticipated to further elucidate the influence of age, genetics, behavior, diet, environment, ecology, and evolution on the gut and oral microbial communities of snakes.

## 1 Introduction

Snakes exhibit diverse diets and live in a variety of habitats, including grasslands, wetlands, forests, agricultural fields, deserts, plateaus, and marine areas (Uetz, 2000). The distinct ecological, physiological, and behavioral characteristics of snakes markedly influence the ecology of their gastrointestinal and oral microbial communities (Costello et al., 2010; Tang et al., 2019). More than 205 snake species are distributed across mainland China (Zhou and Jiang, 2004). However, limited research has been conducted on the microbial communities within these species. *Protobothrops mucrosquamatus*, a medium-sized pitviper characterized by a long triangular head, slender body, and proficiency in tree climbing, is primarily found at lower altitudes in southwest and southeast China, Laos, northern Bangladesh, Vietnam, northern Myanmar, and northeastern India (Zhao, 2006). *Elaphe dione* lives in various habitats, including plains, hills, mountains, grasslands, fields, grass slopes, forests, and riversides, as well as around vegetable gardens, farmhouses, chicken coops, and livestock pens, and mainly feeds on lizards, rats, birds and their eggs, frogs, and insects (Schulz and Entzeroth, 1996). *Gloydius angusticeps*, characterized by its mostly grayish-brown body and short length, rarely exceeding 60 cm, inhabits high-altitude zones in rocky or wetland environments, with a diet primarily consisting of lizards and rats.

Microbial populations within the gastrointestinal tract and oral cavities of both vertebrates and invertebrates play an essential role in nutrient absorption (Mackie, 2002) and can influence host behavior (Archie and Theis, 2011; Ezenwa et al., 2012), immunity (Hooper et al., 2001), reproduction (Shropshire and Bordenstein, 2016), ecology, and evolution. To date, studies on oral microflora have predominantly focused on snakebites (Arroyo et al., 1980; Blaylock, 2001; Campagner et al., 2012; Barbosa et al., 2018; Panda et al., 2019). Furthermore, research on gut microbial communities has been limited to a few species, including *Naja naja* (Krishnankutty et al., 2018), *Ophiophagus hannah* (Krishnankutty et al., 2018), *Python molurus* (Krishnankutty et al., 2018), *Rhabdophis subminiatus* (Tang et al., 2019), Python bivittatus (Costello et al., 2010), *Naja atra*, *Ptyas mucosa*, *Elaphe carinata*, *Deinagkistrodon acutus* (Zhang et al., 2019), and *Ptyas mucosa* (Qin et al., 2019). As such, two critical areas remain to be elucidated, including the ecology of gut and oral bacterial diversity and the interactions between bacterial diversity and factors such as diet, altitude, physiology, and genetics (David et al., 2014; Moeller et al., 2014; McKenney et al., 2015).

Wildlife, noted for their vast diversity and ecological importance, are also reservoirs for a variety of bacterial, viral, and fungal organisms. These microbial communities can include zoonotic pathogens that pose threats to other animals, including rare species. For example, bats and rodents are host to more than 60 zoonotic species (Luis et al., 2013), while wild boars harbor multiple diseases that can threaten rare feline species. However, there is limited information regarding the extent of microbes and the range and nature of diseases potentially harbored by snakes.

To expand our understanding of the gut and oral microbial diversity in *P. mucrosquamatus*, *E. dione*, and *G. angusticeps*, we conducted metagenomic profiling to explore the diversity of bacterial species in fecal and oral samples and to examine the possibility of zoonosis in these snakes.

## 2 Materials and methods

### 2.1 Sample site collection

**FIGURE 1:**
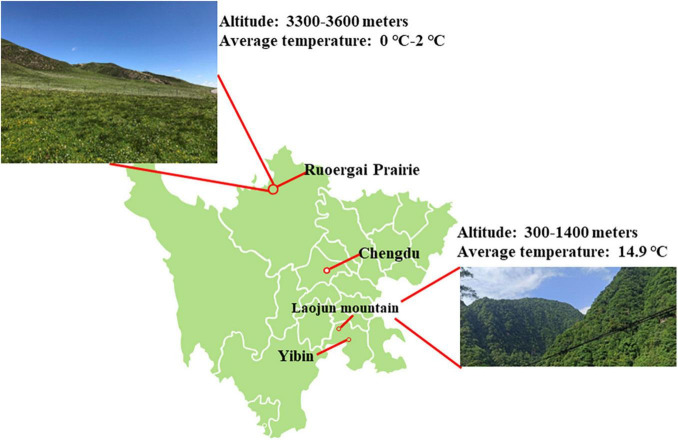
Map of Laojun Mountain nature reserve and Ruoergai Prairie.

The Second Tibetan Plateau Scientific Expedition and Research program included a focus on gut and oral cavity bacterial diversity in reptiles. During July to September 2020, we selected several sampling sites in gullies and forests, capturing four brown-spotted pitvipers (*P. mucrosquamatus*) and three Dione’s rat snakes (*E. dione*) in the Laojun Mountains (28°43′N, 104°04′E) which was Situated west of Yibin city in Sichuan, China (Figure 1). Additionally, three *G. angusticeps* snakes were captured in the grasslands and along the lakeside of Ruoergai Prairie in Sichuan (average altitude of 3 300–3 600 m) (Figure 1). The snakes were captured using specialized tools and the samples were collected and processed as described previously (Tian et al., 2022). Briefly, the three kinds of snakes were individually placed in sterilized tubs and skins of which were cleaned with 75% alcohol to prevent sample contamination. All pharyngeal and anal swab samples were collected opportunistically in areas where snakes were captured. The swabs were placed in RNase-free tubes and immediately transported on dry ice to Shanghai Biozeron Biotechnology Co., Ltd. (China) the same day. The snakes were then released back into the wild. overnight to collect.

### 2.2 DNA extraction

Microbial DNA was extracted from 10 fecal and 10 oral samples in total, respectively, using an EZNA^®^ stool DNA Kit (Omega Bio-tek, Norcross, GA, USA) in accordance with the manufacturer’s protocols. Metagenomic shotgun sequencing libraries were constructed and sequenced at Shanghai Biozeron Biological Technology Co. Ltd. (China). The sequencing reads, after undergoing quality control, were then mapped against the human genome (version: hg19) using the BWA mem algorithm (parameters: -M -k 32 -t 16).^1^ Reads that were filtered to remove host-genome contamination and low-quality data were identified as clean reads and utilized for subsequent analysis.

### 2.3 Read-based phylogenetic annotation

Taxonomic classification of clean reads from each sample was conducted using Kraken2 (Wood and Salzberg, 2014) with a customized Kraken database, which included all bacterial, archaeal, fungal, viral, protozoan, and algal genome sequences in the NCBI RefSeq database (release number: 90). The classification was done at seven phylogenetic levels which are domain, phylum, class, order, family, genus, species, or unclassified. Bracken^2^ was used to calculate the abundance of each taxonomy. The relative abundance at a specific taxonomic level represented the cumulative abundance of all species classified within that level.

### 2.4 Metagenomic *de novo* assembly, gene prediction, and annotation

The clean sequence reads were used to generate a set of contigs for each sample using MegaHit, with the parameter “–min-contig-len 500” (Li et al., 2015). Open reading frames (ORFs) of the assembled contigs were predicted using Prodigal v2.6.3 (Hyatt et al., 2010). Subsequently, the ORFs were clustered with CD-HIT (Fu et al., 2012) to create a unique gene set, with the longest sequence in each cluster serving as the representative sequence for each gene. To calculate gene abundance across all samples, Salmon (Patro et al., 2017) was used to quantify the read count for each gene. Gene abundance was then calculated using the following formulas:

A⁢b⁢(S)=A⁢b⁢(U)+A⁢b⁢(M)

$$A\,b\,\left(U\right)=\sum_{i=1}^{M}1/l$$

$$A\,b\,\left(M\right)=\sum_{i=1}^{M}\left(C\,o*1\right)/l$$

$$C\,o=\frac{A\,b\,\left(U\right)}{\sum_{i=1}^{N}A\,b\,\left(U_{i}\right)}$$

Where *Ab*(*S*) represents gene abundance; *Ab*(*U*) represents single-mapping read abundance; *Ab*(*M*) represents multi-mapping read abundance; and *l* represents length of the gene sequence (Li et al., 2014).

### 2.5 Metagenome-assembled genome (MAG) reconstruction and abundance

Metagenomic binning was applied to the contigs of each sample. The binning process was carried out separately using metaBAT2 (Kang et al., 2015). The completeness and contamination levels of all bins were obtained using CheckM v.1.0.3 (Parks et al., 2015). Bins exhibiting a completeness greater than 50% and contamination less than 10% were classified as “filtered bins.” Assembly quality of the MAGs was enhanced using metaSPAdes (Nurk et al., 2017). All genes within the bins were transformed to protein sequences to generate the proteomes for every bin. These proteomes were used for phylogenetic tree reconstruction using Phylophlan (Segata et al., 2013).

### 2.6 Statistical analysis

Beta diversity was assessed using the Bray-Curtis distance metric to compare the outcomes of principal coordinates analysis (PCoA) using the community ecology package R-forge (Fouts et al., 2012). PCoA was used to explore and visualize data similarities and dissimilarities, starting with a similarity or dissimilarity matrix (= distance matrix) and mapping each item to a location in low-dimensional space to identify the main axes within the matrix. Hierarchical cluster analysis was applied to identify discrete groups with varying degrees of (dis)similarity in a dataset, as represented by a (dis)similarity matrix (like Bray-Curtis distance matrix). These groups were clustered using hierarchical clustering algorithms based on the distance matrix and presented as dendrograms using the unweighted pair-group method with arithmetic averages (UPGMA) (Hou et al., 2019). Heatmaps were generated based on the percentages of microorganisms contained in the matrix, represented as colors using the vegan package in R. Cluster trees of the microorganisms or samples were added to the heatmap analysis to show low- or high-abundant microorganisms in different modules (Fouts et al., 2012). A Venn diagram was drawn using the vegan package in R to analyze overlapping and unique gene sets affecting the bacterial communities during treatment processes (Fouts et al., 2012).

## 3 Results

### 3.1 General characteristics of sequencing data

In this study, we sequenced the fecal and oral metagenomes of 10 captive snakes using the Illumina NovaSeq 6000 platform and obtained more than 200 Gb of high-quality bases, including 290 468 168 raw reads, ranging from 17 737 928 to 39 507 966 reads per sample. The assembled sequences that passed quality control filtering included 2 214 074 contigs, ranging from 50 633 to 761 127 per sample, with an average length of 464–643 bp.

**FIGURE 2:**
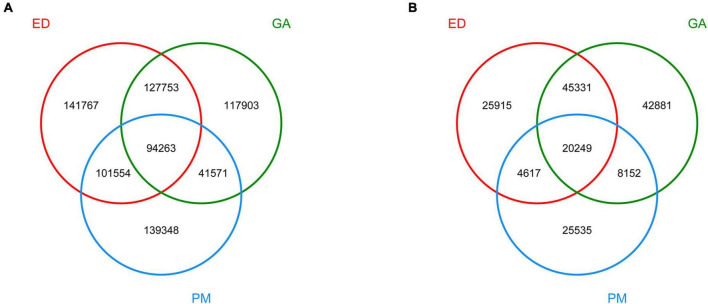
Venn diagram of dereplicated gene set in the fecal **(A)** and oral **(B)** samples of *P. mucrosquamatus*, *E. dione*, and *G. angusticeps*.

Based on Venn diagram analysis, 94 263 and 20 249 contigs were common to all fecal and oral samples, respectively. In addition, 141 767, 119 903, and 139 348 contigs were detected in groups ED, GA, and PM, respectively (Figure 2A), among which 25 915 were unique to ED, 42 881 were unique to GA, and 25 535 were unique to PM (Figure 2B).

### 3.2 Fecal bacterial taxonomic analysis at phyla and genera levels

**FIGURE 3:**
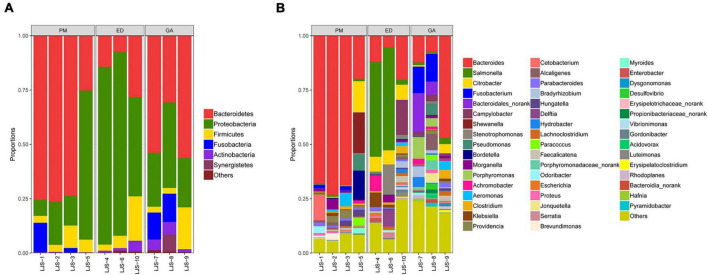
Relative abundance of bacterial communities at the phylum level in fecal **(A)** and oral samples **(B)** in *P. mucrosquamatus*, *E. dione*, and *G. angusticeps.*

Supplementary Table 1 presents the 10 most abundant phyla and genera found in the fecal samples of *P. mucrosquamatus*, *E. dione*, and *G. angusticeps*. Bacteroidetes and Proteobacteria emerged as the dominant phyla (>10% relative abundance) across all species, accounting for 16.69–62.72% and 27.34–70.65% of total contigs, respectively. Firmicutes was the sub-dominant phylum (>1% relative abundance) among the three species, accounting for 5.64–9.66%. In the fecal samples of *G. angusticeps*, the abundances of Fusobacteria and Actinobacteria were notably higher than in the other two species (Figure 3A). Armatimonadetes was also dominant in the guts of all three species, which is reported for the first time.

**FIGURE 4:**
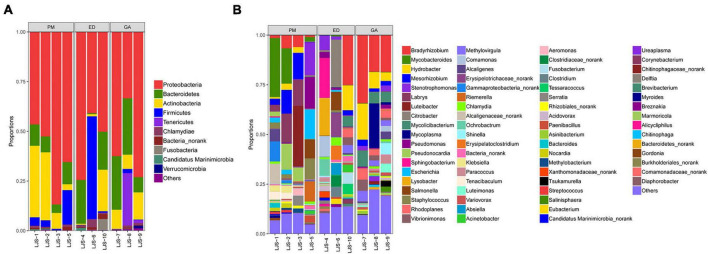
Relative abundance of bacterial communities at the genus level in fecal **(A)** and oral samples **(B)** in *P. mucrosquamatus*, *E. dione*, and *G. angusticeps.*

Among the top 30 bacterial genera identified, 11, 12, and 16 had relative abundances higher than 1% in at least one sample. Among the abundant genera, those showing dominance (>5% relative abundance) in at least one sample included *Bacteroides* (*P. mucrosquamatus*), *Bacteroides*, *Salmonella*, *Citrobacter*, *Campylobacter*, *Stenotrophomonas* (*E. dione*), *Bacteroides*, and *Fusobacterium* (*G. angusticeps*). *Bacteroides* (>20% relative abundance) was the most dominant genus in *P. mucrosquamatus* and *G. angusticeps*, while *Salmonella* (>30% relative abundance) was the most dominant genus in *E. dione* (Figure 4A).

### 3.3 Oral bacterial taxonomic analysis at phylum and genus levels

Proteobacteria was the dominant phylum (>55% relative abundance) in the oral samples of *P. mucrosquamatus*, *E. dione*, and *G. angusticeps*, accounting for 55.16, 56.32, and 62.84% of total contigs, respectively. Bacteroidetes, Actinobacteria, and Firmicutes were the sub-dominant phyla (>7% relative abundance) in the three species, accounting for 8.53–20.98%, 7.53–20.28%, and 0.82–17.54%, respectively. Chlamydiae (>0.68% relative abundance) was detected in all three species, while Tenericutes was detected in *E. dione* and *G. angusticeps* but not in *P. mucrosquamatus* (Figure 3B).

Among the top 30 bacterial genera identified, 16, 17, and 11 had relative abundances higher than 1% in at least one sample. Among the abundant genera, those showing dominance (>5% relative abundance) in at least one sample included *Mycobacteroides*, *Mesorhizobium*, *Labrys*, *Luteibacter*, *Pseudonocardia* (*P. mucrosquamatus*), *Bradyrhizobium*, *Citrobacter*, *Sphingobacterium*, *Lysobacter* (*E. dione*), *Bradyrhizobium*, *Hydrobacter*, *Mycoplasma*, and *Mycolicibacterium* (*G. angusticeps*). *Salmonella* and *Chlamydia* were also detected in the oral samples of all three species, while *Mycoplasma* was present in *E. dione* and *G. angusticeps* but absent in *P. mucrosquamatus* (Figure 4B).

### 3.4 Comparison of bacterial community structure at phylum and genus levels

**FIGURE 5:**
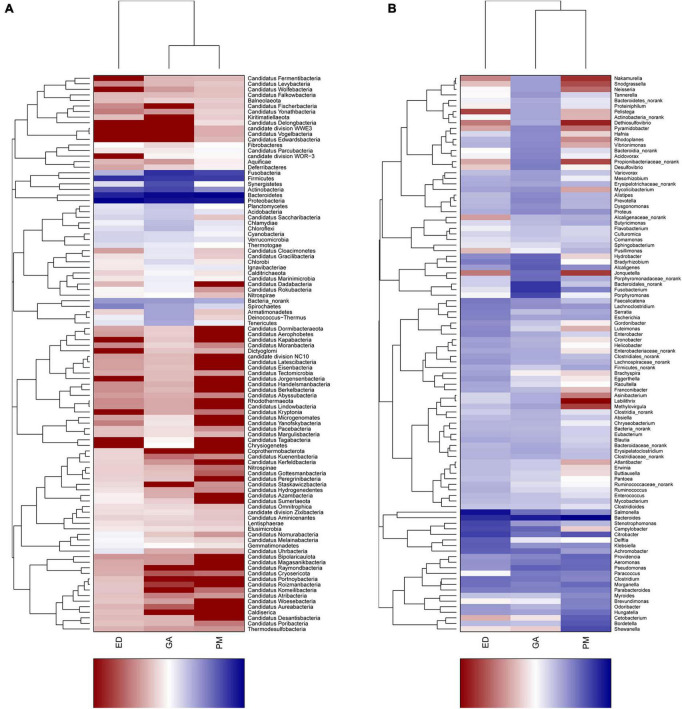
Heatmap showing phylum-level bacterial community composition in fecal **(A)** and oral samples **(B)** of *P. mucrosquamatus*, *E. dione*, and *G. angusticeps*.

Heatmap and abundance analyses revealed distinct bacterial community structures influenced by geographic distribution and species. At the phylum level, Synergistetes, Chlamydiae, Chloroflexi, Chlorobi, Armatimonadetes, Deinococcus-Thermus, and Tenericutes were abundant in the *G. angusticeps* fecal samples (Figure 5A). The phylum Bacteroidetes was significantly more abundant in the fecal samples of *P. mucrosquamatus* than in the two species (Supplementary Table 2). The genus *Salmonella* was significantly more abundant in the *E. dione* fecal samples than in the other two species (Supplementary Table 2). In contrast, Verrucomicrobia, Tenericutes, Planctomycetes, and Chloroflexi were abundant in the *G. angusticeps* oral samples, while Fusobacteria was the only phylum enriched in the oral samples of *E. dione* (Figure 5B).

**FIGURE 6:**
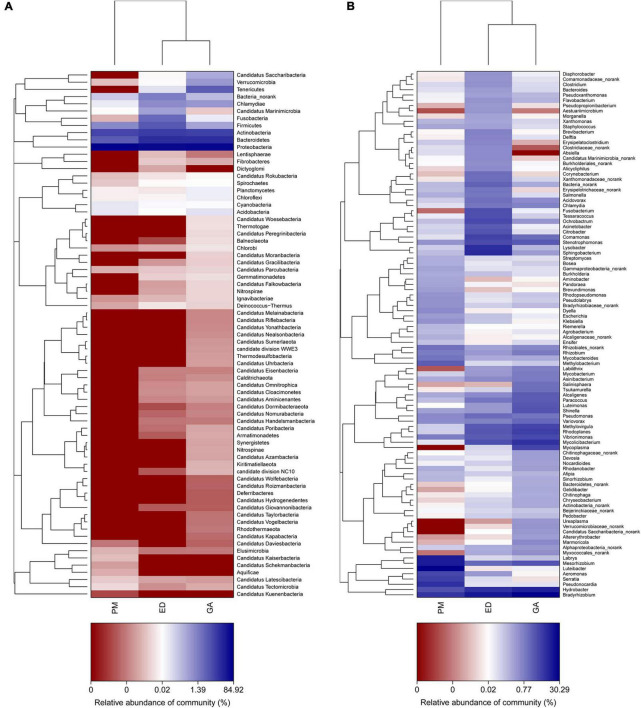
Heatmap showing genus-level bacterial community composition in fecal **(A)** and oral samples **(B)** of *P. mucrosquamatus*, *E. dione*, and *G. angusticeps*.

At the genus level, *Nakamurella*, *Snodgrassella*, *Neisseria*, *Pelistega*, *Dethiosulfovibrio*, *Pyramidobacter*, and *Porphyromonas* were abundant in the fecal samples of *G. angusticeps*, whereas *Delftia* was enriched in the fecal samples of *E. dione* and *Myroides*, *Brevundimonas*, *Cetobacterium*, and *Shewanella* were abundant in the fecal samples of *P. mucrosquamatus* (Figure 6A). *Pseudopropionibacterium*, *Aestuariimicrobium*, *Brevibacterium*, *Corynebacterium*, *Fusobacterium*, *Tessaracoccus*, *Acinetobacter*, *Citrobacter*, *Lysobacter*, and *Diaphorobacter* were abundant in the oral samples of *E. dione*. *Labrys*, *Serratia*, *Pseudonocardia*, *Methylobacterium*, *Aminobacter*, *Pandoraea*, and *Brevundimonas* were enriched in the oral samples of *P. mucrosquamatus*. *Salinisphaera, Tsukamurella*, *Luteimonas*, *Shinella*, *Mycoplasma*, *Ureaplasma*, *Altererythrobacter*, and *Marmoricola* were abundant in oral samples of *G. angusticeps* (Figure 6B). *Mycolicibacterium*, *Rhodoplanes*, and *Methylovirgula* were significantly more abundant in the oral samples of *G. angusticeps* compared to the other two species (Supplementary Table 2).

### 3.5 Comparative analysis of bacterial communities in different sample groups

**FIGURE 7:**
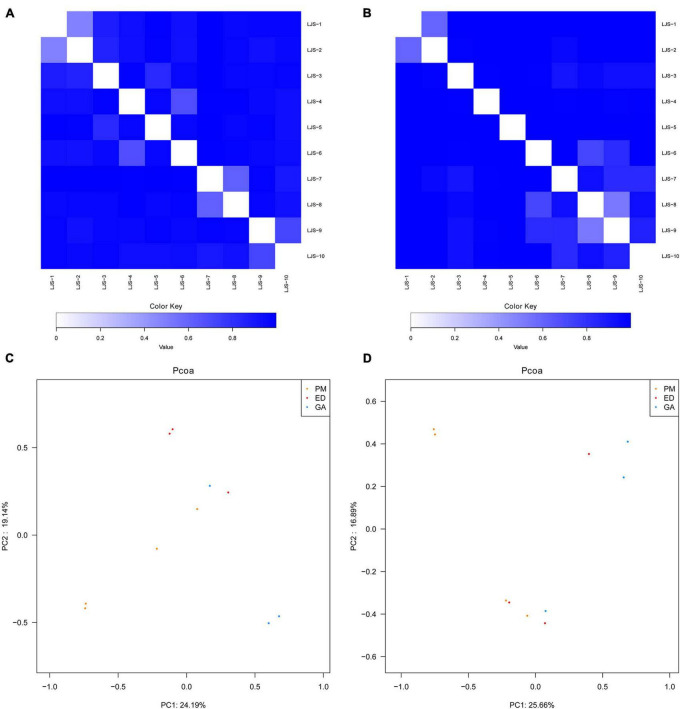
Bray-Curtis distance analysis and principal component analysis of bacterial communities in fecal and oral samples in *P. mucrosquamatus*, *E. dione*, and *G. angusticeps*. Bray-Curtis distance analysis of fecal **(A)** and oral samples **(B)**. Principal component analysis of fecal **(C)** and oral samples **(D)**.

Bray-Curtis distance analysis revealed that bacterial community differences were minor within each species, with samples from the same species clustering together (Figures 7A, B). PCoA demonstrated that gut and oral microbiota from the same host species were more similar to each other than to those of other host species, indicating a higher similarity in gut microbiota within the same snake species (Figures 7C, D).

### 3.6 Bacterial and parasitic pathogens in snakes

A total of 34 zoonotic pathogens were identified in the three snake species studied. Specifically, in *P. mucrosquamatus*, 12 pathogens were found in oral samples and 22 in fecal samples; in *E. dione*, 19 were found in oral samples and 29 in fecal samples; and in *G. angusticeps*, 17 were found in oral samples and 31 in fecal samples. Interestingly, *G. angusticeps* harbored more pathogens than either *E. dione* or *P. mucrosquamatus.* The oral samples from all three snakes contained several pathogens commonly found in urban areas, including *Streptococcus pneumoniae*, *Staphylococcus aureus*, *Mycobacterium tuberculosis*, *Listeria monocytogenes*, *Chlamydia trachomatis*, *Campylobacter jejuni*, and *Salmonella enterica*. *Bartonella* and *Mycoplasma pneumoniae* were only found in the oral samples of *G. angusticeps*, while *Clostridium perfringens*, *Fusobacterium russii*, *Fusobacterium mortiferum*, *Streptobacillus moniliformis*, *Citrobacter freundii*, and *Shigella dysenteriae* were only found in *E. dione.*

## 4 Discussion

Reptiles, a group of vertebrates with ancient origins, encompass over 11 000 species, with more than 30% classified within the snake clade. Despite their considerable diversity, reptiles have been largely overlooked in gut and oral microbiota studies (Uetz, 2000). Few studies have been conducted on the microbial communities of lizards (Hong et al., 2011; Ren et al., 2016; Jiang et al., 2017; Kohl et al., 2017; Zhang et al., 2018; Zhou et al., 2020; Tian et al., 2022; Zhu et al., 2022), snakes (Costello et al., 2010; Krishnankutty et al., 2018; Tang et al., 2019), turtles (Campos et al., 2018), and crocodilians (Keenan et al., 2013; Keenan and Elsey, 2015). Our team has identified the presence of various viral families, such as *Adenoviridae*, *Iflaviridae*, *Circoviridae*, *Retroviridae*, and *Parvoviridae* in the mouths and guts of *P. mucrosquamatus*, *E. dione*, and *G. angusticeps* (Liu et al., 2023).

In the present study, the most dominant phyla found in the fecal samples of *P. mucrosquamatus*, *E. dione*, and *G. angusticeps* were Bacteroidetes (62.7, 16.7, and 46.9%), Proteobacteria (27.3, 70.7, and 29.0%), and Firmicutes (5.6, 9.7, and 8.2%), collectively accounting for 84.1% of the sequences in the three snake species. Previous studies have also reported that presence of Firmicutes (61.8%), Bacteroidetes (20.6%), and Proteobacteria (10.1%) in Burmese pythons (Costello et al., 2010) and Proteobacteria (65.3%), Firmicutes (9.5%), and Bacteroidetes (9.03%) in *Rhabdophis subminiatus* (Tang et al., 2019). In our study, Bacteroidetes showed higher abundance in two of the three Chinese snakes (*P. mucrosquamatus* and *G. angusticeps*), but relatively lower abundance in Burmese pythons and *R. subminiatus.* The phylum Bacteroidetes, known to be abundant in many mammalian gut communities, exhibits lower abundance in insectivorous mammals (Ley et al., 2008; Shinohara et al., 2019), consistent with our previous findings in lizards (Tian et al., 2022). Further investigation is required to clarify the role of Bacteroidetes in snake evolution, behavior, and digestion. Proteobacteria, commonly present in domestic insectivorous lizards, is thought to enhance cellulose activity and promote nutrient absorption in hosts (Colston and Jackson, 2016; Tian et al., 2022). However, whether the abundance of Proteobacteria in *E. dione* and *R. subminiatus* is related to diet remains unclear. Interestingly, the dominance of Armatimonadetes in the three species, the first report of such, suggests a potential symbiotic role in degrading C5 sugars in hemicelluloses (Lee et al., 2014). Among the Chinese snakes, *G. angusticeps* exhibited a more diverse bacterial composition with seven abundant phyla compared to *P. mucrosquamatus* and *E. dione.* This may be attributed to *G. angusticeps* inhabiting high-altitude regions with minimal human contact, suggesting that unique habitats may influence gut microbial composition. However, the small sample size of snakes used in this study may introduce bias, warranting caution in interpreting these results. Overall, these findings provide new insights into the bacterial composition of reptiles from diverse habitats, particularly differences between high- and low-altitude environments, laying the groundwork for future research regarding the impact of altitude on bacterial diversity in reptiles.

Firmicutes, Actinobacteria, Proteobacteria, and Bacteroidetes are commonly found in the oral bacterial compositions of various species, including humans, mice, felines, canines, chimpanzees, hawks, and lizards (Chun et al., 2010; Sturgeon et al., 2013; Gong et al., 2014; Dewhirst et al., 2015; Adler et al., 2016; Tian et al., 2022). However, research on the oral microbiota of snakes remains limited. In the present study, Proteobacteria (62.8, 55.2, and 56.3%), Bacteroidetes (8.5, 13.9, and 21.0%), Actinobacteria (20.2, 7.5, and 10.2%), and Firmicutes (6.4, 17.5, and 0.8%) were also prevalent in the oral samples of *P. mucrosquamatus*, *E. dione*, and *G. angusticeps*, indicating a bacterial composition similar to the abovementioned hosts. Verrucomicrobia, Tenericutes, Planctomycetes, and Chloroflexi were more abundant in *G. angusticeps* compared to *P. mucrosquamatus* and *E. dione.* Fusobacteria was particularly enriched in the oral samples of *E. dione.* Interestingly, Tenericutes, known for playing a key role in the gut communities of fish and juvenile amphibians (Colston and Jackson, 2016), was also enriched in *G. angusticeps*. Moreover, the genera *Mycolicibacterium*, *Rhodoplanes*, and *Methylovirgula* were markedly more abundant in *G. angusticeps* than in *P. mucrosquamatus* and *E. dione* (Table 1). *Rhodoplanes* is broadly distributed in aquatic habitats (Srinivas et al., 2014), while *Methylovirgula* is widely distributed in forest soils and acidic wetlands (pH 3–5) (Vorob’ev et al., 2009) and *Mycolicibacterium* is known to thrive in acid-resistant environments (Xia et al., 2022). These findings indicate that the oral environment of *G. angusticeps* may be more acidic than that of the other two species, consistent with its habits and wetland environment. Overall, our study suggests that oral bacterial composition varies in different snakes based on geography, diet, and habitat.

**TABLE 1:** The differences in relative abundance (% ± SD) of the abundant phylum and genera of three snake species.

Fecal samples	*P. mucrosquamatus*		*E. dione*		*G. angusticeps*	
Phyla	Mean	SD	Mean	SD	Mean	SD
Bacteroidetes	62.71718^a^	25.00718	16.69225^a^	10.53922	46.93129	14.1189
Proteobacteria	27.34242	27.95013	70.65493	21.66673	28.96188	9.074718
Firmicutes	5.645103	3.365081	9.660418	9.466791	8.208084	9.631486
Fusobacteria	3.897432	6.491925	0.05476439	0.0418832	8.52378	7.293321
**Genera**	**Mean**	**SD**	**Mean**	**SD**	**Mean**	**SD**
Bacteroides	57.53221	25.1977	12.57475	7.48112	22.24819	21.51843
Salmonella	0.5799507^a^	0.4822157	31.07246^a^	25.04976	1.570575	1.184337
Citrobacter	3.757409	7.062554	6.795001	0.2339335	1.689621	2.271929
Fusobacterium	0.9225616	1.064544	0.04304563	0.03522231	8.401912	7.20272
**Oral samples**	***P. mucrosquamatus***		***E. dione***		***G. angusticeps***	
**Phyla**	**Mean**	**SD**	**Mean**	**SD**	**Mean**	**SD**
Proteobacteria	62.84256	17.8115	55.15867	17.35485	56.31606	20.52957
Bacteroidetes	8.530153	3.162945	13.88873	11.65967	20.98169	11.55631
Actinobacteria	20.27711	17.23851	7.527482	11.5808	10.18887	3.293486
Firmicutes	6.408648	7.466746	17.54082	29.60307	0.8170944	1.144098
**Genera**	**Mean**	**SD**	**Mean**	**SD**	**Mean**	**SD**
Bradyrhizobium	3.789806	2.984883	8.425448	14.47979	23.75368	9.074653
Mycolicibacterium	0.09277911^bc^	0.05401312	1.780452^c^	3.040873	6.357453^b^	1.469326
Rhodoplanes	0.06965061^b^	0.02688067	1.485428	2.561949	4.054648^b^	1.299689
Methylovirgula	0.08504497^b^	0.05328704	1.333256	2.295756	3.578454^b^	1.460399

^a^Indicates the values with significant differences in the relative abundance of Bacteroidetes between *P. mucrosquamatus* and *E. dione* in fecal samples (*P* < 0.05).

^b^Indicates the values with significant differences in the relative abundance of Mycolicibacterium, Rhodoplanes, Methylovirgula between *P. mucrosquamatus* and *G. angusticeps* (*P* < 0.05).

^c^Indicates the values with significant differences in the relative abundance of Mycolicibacterium between *P. mucrosquamatus* and *E. dione* (*P* < 0.05). SD, standard deviation.

Limited microbiological data exist regarding wound infections from snakebites. However, understanding the origin of bacteria in the mouth of snakes is crucial given the differences in bacterial infection severity from the bites of certain species, such as the cobra, relative to those from other snakes (Mao et al., 2016). In the current study, we identified a total of 34 bacterial and parasitic pathogens in the three Chinese snake species, some of which were zoonotic. Notably, 10 pathogenic species (*Clostridium botulinum*, *Streptococcus pneumoniae*, *Pseudomonas aeruginosa*, *Comamonas testosteroni*, *Staphylococcus aureus*, *Mycobacterium tuberculosis*, *Listeria monocytogenes*, *Chlamydia trachomatis*, *Campylobacter jejuni*, and *Salmonella enterica*) were detected in the oral samples of all three snakes, while more than 24 pathogens (*Clostridium perfringens*, *Clostridium baratii*, *Clostridium botulinum*, *Fusobacterium russii*, *Fusobacterium mortiferum*, *Prevotella loescheii*, *Enterococcus durans*, *Streptococcus pneumoniae*, *Citrobacter freundii*, *Pseudomonas aeruginosa*, *Comamonas testosterone*, *Enterococcus cecorum*, *Staphylococcus aureus*, *Mycobacterium tuberculosis*, *Listeria monocytogenes*, *Chlamydia trachomatis*, *Yersinia pseudotuberculosis*, *Yersinia enterocolitica*, *Campylobacter jejuni*, *Campylobacter coli*, *Salmonella enterica*, *Leptospira*, *Bartonella*, *Shigella dysenteriae*) were found in the gut samples of all three snakes. Various wild animals, such as rodents, boars, bats, and birds, are known carriers of many pathogens, posing considerable disease risk to both other wildlife and humans (Benskin et al., 2009; Selvin et al., 2019; Liu et al., 2022; Tapia-Ramírez et al., 2022). Therefore, the role of snakes as potential vectors of zoonosis is an important area of focus.

*Bartonella*, an increasing recognized vector-borne pathogen, infects a variety of hosts including humans, foxes (Kosoy and Goodrich, 2018), wolves (Greco et al., 2021), badgers (Bitam et al., 2009), hedgehogs (Bitam et al., 2009), roe deer (Dehio et al., 2001), and canids (Gerrikagoitia et al., 2012). This study marks the first identification of *Bartonella* in the gastrointestinal tract of the three snake species, as well as in the oral cavity of *G. angusticeps.* Given that *Bartonella* species are transmitted by arthropods, these observations underscore the need for more targeted research into the interactions between snakes and arthropod vectors.

In conclusion, metagenomic analysis of fecal and oral samples from three Chinese snake species revealed a diverse bacterial composition, dominated by Bacteroidetes, Proteobacteria, and Firmicutes in the fecal samples and by Proteobacteria, Bacteroidetes, Actinobacteria, and Firmicutes in oral samples across all three species. Notably, however, our results also showed that the microbiological data from *G. angusticeps*, which inhabits high-altitude regions, differed significantly from those of *P. mucrosquamatus* and *E. dione*, which reside in low-altitude regions. Furthermore, a variety of bacterial pathogens were identified in both the oral and fecal samples, which resulted in the secondary infections of bite wounds and was transmitted to the other wild field animals, suggesting that snakes may serve as a natural reservoir of zoonotic diseases. Future research should focus on (1) exploring how gut and oral microbial communities influence the ecology and evolution of snakes, and (2) investigating interactions between snakes and wildlife, including arthropods, birds, rodents, and bats, to better understand the transmission of emerging infectious diseases.

## Data availability statement

The datasets presented in this study can be found in online repositories. The names of the repository/repositories and accession number(s) can be found below: NCBI–SRP406182.

## Ethics statement

The animal study was approved by the Committee on the Animal Ethics Procedures and Guidelines of Experiment Animals Committee affiliated with Yibin University, Yibin, China. The study was conducted in accordance with the local legislation and institutional requirements.

## Author contributions

XH: Funding acquisition, Writing – original draft, Writing – review and editing. LY: Software, Writing – original draft. YZ: Software, Writing – original draft. MY: Investigation, Writing – original draft. JL: Formal analysis, Writing – original draft. YF: Methodology, Writing – original draft. PG: Methodology, Writing – review and editing. ZT: Data curation, Funding acquisition, Writing – review and editing.

## Footnotes

1
http://bio-bwa.sourceforge.net/bwa.shtml

2
https://ccb.jhu.edu/software/bracken/
