# Supplementary material for: Fecal and oral microbiome analysis of snakes from China reveals a novel natural emerging disease reservoir
Source: Front Microbiol. 2024 Jan 11;14:1339188. doi: 10.3389/fmicb.2023.1339188 (PMC10808610; doi:10.3389/fmicb.2023.1339188)
Supplement: Supplementary file 1 [file Table_1.DOCX]

Table 1: the list of bacterial and parasite pathogens in snakes

| **type** | **Oral** | | | | **Feces** | | | | **Oral** | | | **Feces** | | | **Oral** | | | **Feces** | | |
| --- | --- | --- | --- | --- | --- | --- | --- | --- | --- | --- | --- | --- | --- | --- | --- | --- | --- | --- | --- | --- |
|  | ***Protobothrops mucrosquamatus*** | | | | | | | | *Elaphe dione* | | | | | | *Gloydius angusticeps* | | | | | |
|  | **LJS-1** | **LJS-2** | **LJS-3** | **LJS-5** | **LJS-1** | **LJS-2** | **LJS-3** | **LJS-5** | **LJS-4** | **LJS-6** | **LJS-10** | **LJS-4** | **LJS-6** | **LJS-10** | **LJS-7** | **LJS-8** | **LJS-9** | **LJS-7** | **LJS-8** | **LJS-9** |
| ***Clostridium perfringens*** |  |  |  |  | **✔** | **✔** | **✔** | **✔** |  | **✔** |  | **✔** | **✔** | **✔** |  |  |  | **✔** | **✔** | **✔** |
| ***Clostridium baratii*** |  |  |  |  | **✔** | **✔** | **✔** | **✔** |  | **✔** |  | **✔** | **✔** | **✔** |  | **✔** |  |  |  | **✔** |
| ***Clostridium botulinum*** | **✔** | **✔** | **✔** |  | **✔** | **✔** | **✔** | **✔** |  | **✔** | **✔** | **✔** | **✔** | **✔** |  |  | **✔** | **✔** | **✔** | **✔** |
| ***Fusobacterium russii*** |  |  |  |  | **✔** |  | **✔** |  |  |  | **✔** |  | **✔** |  |  |  |  | **✔** | **✔** |  |
| ***Fusobacterium mortiferum*** |  |  |  |  | **✔** | **✔** | **✔** |  |  |  | **✔** |  | **✔** | **✔** |  |  |  | **✔** | **✔** | **✔** |
| ***Prevotella denticola*** |  |  |  |  |  |  |  | **✔** |  |  |  |  | **✔** |  |  |  |  |  |  |  |
| ***Prevotella melaninogenica*** |  |  |  |  |  |  |  |  |  |  |  |  |  |  |  |  |  | **✔** | **✔** |  |
| ***Prevotella loescheii*** |  |  |  |  | **✔** |  |  |  |  |  |  |  |  | **✔** |  |  |  |  | **✔** |  |
| ***Enterococcus durans*** |  |  |  |  | **✔** |  | **✔** |  |  |  |  |  | **✔** | **✔** |  |  |  | **✔** |  |  |
| ***Streptococcus minor*** |  |  |  |  |  |  |  |  |  |  |  |  |  |  |  |  |  |  | **✔** |  |
| ***Streptococcus pneumoniae*** | **✔** | **✔** | **✔** |  |  |  | **✔** | **✔** |  | **✔** |  | **✔** | **✔** | **✔** |  |  | **✔** | **✔** | **✔** | **✔** |
| ***Streptobacillus moniliformis*** |  |  |  |  |  |  |  |  |  |  | **✔** |  |  |  |  |  |  |  |  |  |
| ***Citrobacter freundii*** |  |  |  |  |  | **✔** | **✔** | **✔** |  |  | **✔** | **✔** | **✔** | **✔** |  |  |  | **✔** | **✔** | **✔** |
| ***Lactococcus garvieae*** |  |  |  |  |  | **✔** |  |  |  |  |  |  | **✔** | **✔** |  |  |  |  |  |  |
| ***Pseudomonas aeruginosa*** | **✔** |  | **✔** |  | **✔** | **✔** | **✔** | **✔** | **✔** |  | **✔** | **✔** | **✔** | **✔** | **✔** | **✔** | **✔** | **✔** | **✔** | **✔** |
| ***Comamonas testosteroni*** | **✔** |  | **✔** |  | **✔** | **✔** | **✔** | **✔** | **✔** |  | **✔** | **✔** | **✔** | **✔** |  |  | **✔** | **✔** | **✔** | **✔** |
| ***Enterococcus cecorum*** |  |  |  |  |  | **✔** | **✔** | **✔** |  |  |  |  |  | **✔** |  |  |  |  | **✔** | **✔** |
| ***Staphylococcus aureus*** | **✔** | **✔** | **✔** | **✔** |  |  |  | **✔** | **✔** | **✔** | **✔** | **✔** | **✔** | **✔** |  | **✔** |  | **✔** |  | **✔** |
| ***Mycobacterium tuberculosis*** | **✔** | **✔** | **✔** |  | **✔** | **✔** | **✔** | **✔** | **✔** | **✔** | **✔** | **✔** | **✔** | **✔** |  | **✔** | **✔** | **✔** | **✔** | **✔** |
| ***Listeria monocytogenes*** |  |  | **✔** |  | **✔** | **✔** | **✔** | **✔** |  | **✔** | **✔** | **✔** | **✔** | **✔** | **✔** |  |  | **✔** | **✔** | **✔** |
| ***Chlamydia trachomatis*** | **✔** |  | **✔** |  | **✔** |  | **✔** |  |  | **✔** | **✔** | **✔** |  | **✔** | **✔** | **✔** | **✔** | **✔** | **✔** | **✔** |
| ***Desulfovibrio desulfuricans*** |  |  |  |  | **✔** |  | **✔** |  |  |  |  |  |  |  |  |  |  |  | **✔** |  |
| ***Mycoplasma pneumoniae*** |  |  |  |  |  |  |  |  |  |  |  |  |  |  |  | **✔** | **✔** | **✔** |  |  |
| ***Yersinia pseudotuberculosis*** |  |  | **✔** |  | **✔** | **✔** |  |  |  |  |  | **✔** | **✔** | **✔** |  |  | **✔** | **✔** | **✔** | **✔** |
| ***Yersinia enterocolitica*** |  |  |  |  | **✔** | **✔** | **✔** |  |  | **✔** |  | **✔** | **✔** | **✔** | **✔** |  |  | **✔** | **✔** | **✔** |
| ***Campylobacter jejuni*** | **✔** | **✔** | **✔** | **✔** |  | **✔** |  |  |  | **✔** |  | **✔** |  | **✔** |  | **✔** |  | **✔** | **✔** | **✔** |
| ***Campylobacter coli*** | **✔** |  |  |  |  | **✔** |  |  |  |  |  | **✔** | **✔** | **✔** |  | **✔** |  | **✔** | **✔** | **✔** |
| ***Salmonella enterica*** | **✔** |  | **✔** | **✔** | **✔** | **✔** | **✔** | **✔** | **✔** | **✔** | **✔** | **✔** | **✔** | **✔** | **✔** | **✔** | **✔** | **✔** | **✔** | **✔** |
| ***Pasteurella multocida*** |  |  |  |  |  |  |  |  |  |  |  | **✔** | **✔** |  |  |  |  | **✔** | **✔** | **✔** |
| ***Leptospira*** |  |  |  |  | **✔** | **✔** |  | **✔** |  |  | **✔** |  | **✔** | **✔** | **✔** |  |  | **✔** | **✔** |  |
| ***Bartonella*** |  |  |  |  | **✔** | **✔** |  |  |  |  |  | **✔** | **✔** | **✔** |  | **✔** |  |  | **✔** |  |
| ***Borrelia*** |  |  |  |  |  |  | **✔** |  |  |  |  |  |  |  |  |  |  |  | **✔** |  |
| ***Shigella dysenteriae*** |  |  |  |  | **✔** | **✔** | **✔** | **✔** |  | **✔** |  | **✔** | **✔** | **✔** |  |  |  | **✔** |  | **✔** |
